# Supplementary material for: The Role of ERG11 Point Mutations in the Resistance of Candida albicans to Fluconazole in the Presence of Lactate
Source: Pathogens. 2022 Nov 3;11(11):1289. doi: 10.3390/pathogens11111289 (PMC9698267; doi:10.3390/pathogens11111289)
Supplement: Supplementary file 1 [file pathogens-11-01289-s001.zip › pathogens-1919014-supplementary.pdf]

Supplementary Material

The role of *ERG11* point mutations in the resistance of *Candida albicans* to fluconazole in the presence of lactate

Aneta K. Urbanek [1,#,] *, Zofia Łapińska [1,2,#], Daria Derkacz [1] and Anna Krasowska [1]

[1] University of Wroclaw, Faculty of Biotechnology, 50-383 Wroclaw, Poland
[2] Department of Molecular and Cellular Biology, Faculty of Pharmacy, Wroclaw Medical University, Borowska 211A, 50-556 Wroclaw
* Correspondence: aneta.urbanek@uwr.edu.pl
#Aneta K. Urbanek and Zofia Łapińska contributed equally to this work

Table S1. The percentage reduction in the growth of C. albicans CAF2-1 after FLC treatment in presence of YPD and YPL.

	FLC [µg/mL]								
	**0**	**0.125**	**0.25**	**0.5**	**1**	**2**	**4**	**8**	**16**
					**YPD**				
**OD**	1.118	1.133	1.143	1.113	1.008	0.878	0.705	0.624	0.636
**reduction in growth [%]**	-	101.402 ±1,081	102.230 ±1,628	**99.582** ±2.457	**90.158** ±0.896	**78.527** ±3.729	**63.048** ±2.005	**55.860** ±1.735	**56.904** ±3.817
					**YPL**				
**OD**	0.460	0.426	0.459	0.393	0.251	0.211	0.228	0.210	0.180
**reduction in growth [%]**	-	93.422 ±11.806	100.722 ±13.637	**86.838** ±19.897	**55.257** ±9.811	**45.629** ±9.682	**50.134** ±8.183	**46.475** ±15.372	**39.121** ±7.053

Table S2. Identification of amino acid substitutions in *hot spots* as a result of aligning the amino acid sequences of the Erg11p of *Candida* species with the sequence of the Erg11p of *C. albicans* SC5314 strain. "." - no mutation.

				Hot spot **I**														**II**							**III**																											
**Isolate**	**Organism**	**GenBank Number**	**MIC [μg/mL] FLC**	**F105**	**S110**	**D116**	**K119**	**K128**	**Y132**	**S137**	**S142**	**F148**	**D153**	**S154**	**K156**	**K161**	**R163**	**E266**	**R267**	**D271**	**N273**	**L281**	**I282**	**H283**	**H410**	**S412**	**E413**	**R414**	**Y415**	**F416**	**D417**	**N418**	**A421**	**D423**	**W427**	**D428**	**T429**	**A434**	**N435**	**S436**	**S442**	**V452**	**S453**	**E473**	**V478**	**I483**	**L484**	**T485**	**F487**	**V488**	**Source**
SC5314	*C.albicans*	AY856352	0.25	.	.	.	.	.	.	.	.	.	.	.	.	.	.	.	.	.	.	.	.	.	.	.	.	.	.	.	.	.	.	.	.	.	.	.	.	.	.	.	.	.	.	.	.	.	.	.	
CBS 10913	*C. auris*	MK059959.1	2	L	A	A	S	.	.	.	.	T	E	A	Q	R	Q	K	T	V	D	M	T	N	Q	N	.	K	W	.	.	.	E	.	.	.	.	.	.	.	T	.	T	.	.	.	.	.	Y	.	[50]
C54019	*C. auris*	MK059964.1	> 32	L	A	A	S	.	.	.	R	T	E	A	Q	.	Q	K	T	V	D	M	T	N	Q	N	.	K	W	.	.	R	.	.	.	.	.	.	.	.	T	.	T	.	.	.	.	.	Y	.	[50]
CD36	*C. dubliniensis*	XP_002420370	0.25	.	.	.	.	.	.	.	.	.	E	.	.	.	.	.	.	.	S	.	T	.	.	.	.	.	.	.	.	.	N	.	.	.	.	.	.	.	.	I	.	.	.	.	.	.	.	.	[51]
NCPF 3949	*C. dubliniensis*	AYO34876	0.125	.	.	.	.	.	.	.	.	.	E	.	.	.	.	.	.	.	.	.	.	.	.	.	.	.	.	.	.	.	.	.	.	.	.	.	.	.	.	I	.	.	.	.	.	.	.	.	[52]
CBS 138	*C. glabrata*	AAB02329.1	> 32	.	A	.	.	.	.	.	.	.	E	A	.	.	.	.	K	.	.	M	.	N	.	.	.	.	F	.	.	.	E	N	.	.	.	S	S	A	.	I	.	.	.	L	M	S	.	I	[52]
CBS 860	*C. glabrata*	AAXO2335.1	≥ 64	.	A	.	.	.	.	.	.	.	E	A	.	.	.	.	K	.	.	M	V	N	.	.	.	.	F	.	.	.	E	N	.	.	.	S	S	A	.	I	.	.	.	L	.	S	.	I	[52]
B2455	*C. kefyr*	AHL25033.1	> 320	.	A	.	.	E	.	.	.	.	.	A	R	L	.	.	.	.	D	M	.	.	.	.	D	.	.	.	.	.	E	N	.	.	.	.	.	.	.	I	.	.	M	L	.	.	.	.	[54]
B2399	*C. krusei*	ABY26732.1	160	.	.	.	.	.	.	.	.	.	.	.	.	.	.	.	.	.	.	.	.	.	.	T	Q	.	.	.	.	.	N	N	F	.	.	.	.	N	.	.	.	D	I	.	.	.	.	.	[54]
Co690	*C. orthopsilosis*	MG543289.1	1	.	.	.	Q	.	.	A	.	T	.	.	R	L	.	.	S	.	.	.	V	N	.	N	.	.	F	Y	.	D	.	N	.	.	.	.	.	.	A	.	.	.	.	.	.	.	.	.	[38]
Rome24	*C. orthopsilosis*	AWB13383.1	128	.	.	.	Q	.	F	A	.	.	.	.	R	.	.	.	.	.	.	.	V	N	.	N	.	.	F	Y	.	D	.	N	.	.	.	.	.	.	A	.	.	.	.	.	.	.	.	.	[38]
ATCC 22019	*C. parapsilosis*	ACT67904.1	0.15	.	.	.	Q	.	.	A	.	.	.	.	R	.	.	.	.	.	.	.	V	N	.	N	.	.	F	Y	.	D	.	N	.	.	.	.	.	.	A	.	.	.	.	.	.	.	.	.	[55]
Kw 2097-14	*C. parapsilosis*	SBU87516.1	64	.	.	.	Q	.	F	A	.	.	.	.	R	.	.	.	.	.	.	.	V	N		N	.	.	F	Y	.	D		N	.	.	.	.	.	.	A	.	.	.	.	.	.	.	.	.	[56]
ATCC 750	*C. tropicalis*	AAA53284.1	1	Y	.	E	.	.	.	.	.	.	.	.	.	.	.	.	.	.	.	.	V	N	.	.	D	.	W	.	E	H	.	N	.	W	S	.	S	A	.	I	.	.	.	.	.	.	Y	I	[57]
IHEM 21234	*C. tropicalis*	AAX39315.1	≥ 256	Y	.	E	.	.	F	.	.	.	.	.	.	.	.	.	.	.	.	.	V	N	.	.	D	.	W	.	E	H	.	N	.	E	S	.	S	A	.	I	.	.	.	.	.	.	Y	I	[41]

Table S3. Identification of amino acid substitutions in *hot spots* as a result of aligning the amino acid sequences of the Erg11p *C. albicans* strains with the amino acid sequence of the Erg11p of *C. albicans* SC5314 strain. "." - no mutation.

			Hot spot																						
			I										II		III										
**Isolate**	**GenBank Number**	**MIC [µg/mL] FLC**	**A114**	**D116**	**T123**	**F126**	**K128**	**G129**	**Y132**	**N136**	**K143**	**V159**	**E266**	**T285**	**V437**	**N440**	**G448**	**G450**	**V456**	**S457**	**G464**	**G465**	**R467**	**V488**	**Source**
**SC5314**	AY856352	0.25	.	.	.	.	.	.	.	.	.	.	.	.	.	.	.	.	.	.	.	.	.	.	
**CAAL - 9**	EU885928.1	< 0.125	.	.	.	.	.	.	.	.	.	.	D	.	.	.	.	.	.	.	.	.	.	I	[35]
**CAAL - 11**	EU885930.1	< 0.125	.	E	.	.	T	.	.	.	.	.	.	.	.	.	.	.	.	.	.	.	.	.	[35]
**CAAL - 15**	EU885934.1	< 0.125	.	E	.	.	T	.	.	.	.	I	.	.	.	.	.	.	.	.	.	.	.	.	[35]
**CAAL - 37**	EU885935.1	> 64	.	.	.	.	.	.	.	.	.	.	D	.	.	.	.	.	I	.	S	.	.	I	[35]
**CAAL - 62**	EU885937.1	0.125	.	E	.	.	T	.	.	.	.	.	.	.	.	.	.	.	.	.	.	.	.	.	[35]
**CAAL-70**	EU885938.1	16	.	.	.	.	.	.	H	Y	.	.	.	.	.	.	.	.	.	.	.	.	.	.	[35]
**CHC34**	KM609916	8	.	.	.	.	.	.	.	.	.	.	.	.	.	.	.	.	.	.	.	.	.	.	[58]
**CHC95**	KM609917	16	.	.	.	.	.	.	H	.	.	.	D	.	.	S	.	.	.	P	.	.	.	I	[58]
**CHC106**	KM609918	16	.	.	.	.	.	.	.	.	.	.	.	.	I	.	.	.	.	.	.	.	.	.	[58]
**CHC114**	KM609920	> 64	E	.	.	.	.	.	.	.	.	.	.	.	.	.	.	.	.	.	.	.	.	.	[58]
**CHC117**	KM609921	> 64	.	E	.	.	T	.	H	.	.	.	.	.	.	.	.	.	.	.	.	S	.	.	[58]
**CHC119**	KM609922	> 64	.	.	I	.	.	.	H	.	.	.	.	.	.	.	.	.	.	.	.	.	.	.	[58]
**CHC122**	KM609924	> 64	S	.	.	.	.	.	.	.	.	.	.	.	.	.	.	.	.	.	.	.	.	.	[58]
**CHC125**	KM609925	> 64	.	.	I	.	.	.	H	.	.	.	.	A	.	.	.	.	.	.	.	.	.	.	[58]
**CHC126**	KM609926	32	.	.	.	.	.	.	.	.	.	.	D	.	.	.	.	.	.	.	.	.	.	.	[58]
**CHC128**	KM609927	> 64	S	.	.	.	.	.	.	.	.	.	.	.	.	.	.	.	.	.	.	.	.	.	[58]
**CHC130**	KM609928	> 64	S	.	.	.	.	.	.	.	.	.	.	.	.	.	.	.	.	.	.	.	.	.	[58]
**CHC134**	KM609929	> 64	.	E	.	.	T	.	.	.	.	.	.	.	.	.	.	.	.	.	S	.	.	.	[58]
**CHC157**	KM609930	> 64	S	.	.	.	.	.	.	.	.	.	.	.	.	.	.	.	.	.	.	.	.	.	[58]
**CHC160**	KM609931	> 64	.	E	.	.	T	.	H	.	.	.	.	.	.	.	.	.	.	.	.	S	.	.	[58]
**P94015**	KGQ85038.1	>64	.	.	.	L	.	.	.	.	.	.	.	.	.	.	.	.	.	.	.	.	K	.	[59]
**10C1B1M1**	KM875722.1	16	.	.	.	.	.	.	.	.	R	.	.	.	.	.	.	.	.	.	.	.	.	.	[7]
**20E1II1G1**	KM875728.1	256	.	.	.	.	.	.	F	.	.	.	.	.	.	.	.	.	.	.	.	.	.	.	[7]
